# Utility of contrast enhanced ultrasound (CEUS) in penile trauma

**DOI:** 10.1186/s13244-023-01499-2

**Published:** 2023-09-25

**Authors:** Miguel A. Gómez-Bermejo, Dean Y. Huang, Michele Bertolotto, Paul S. Sidhu

**Affiliations:** 1 Department of Radiology, Ramón y Cajal University Hospital, Colmenar Viejo Street, km9, 28034 Madrid, Spain; 2 Department of Imaging Sciences, School of Biomedical Engineering and Imaging Sciences, Faculty of Life Sciences and Medicine, King’s College London, London, SE17EH UK; 3 Department of Radiology, King’s College London, King’s College Hospital NHS Foundation Trust, Denmark Hill, London, SE5 9RS UK; 4 Department of Radiology, Hospital of Cattinara, University of Trieste, Trieste, Italy

**Keywords:** Contrast enhanced ultrasound, Penile trauma, Penile fracture, Penile haematoma

## Abstract

Penile traumatic injuries are rare with potentially severe consequences, with clinical assessment challenging due to pain and swelling. Ultrasound is the primary imaging modality for assessing the penis. It has a high spatial resolution and rapid availability and is portable. Contrast enhanced ultrasound is an advanced ultrasound technique that adds real-time dynamic vascular imaging safely, increasing diagnostic precision and confidence, allowing for better therapeutic management of traumatic penile injuries. Contrast enhanced ultrasound helps to delineate the extent of a haematoma, distinguishing this from the herniated corpus cavernosum, detecting the point of rupture of the tunica albuginea, showing vascular abnormalities, localising a dislocated penis and determining penile viability. The integrity of the urethra may be assessed with intracavitary contrast enhanced ultrasound. Additionally, contrast enhanced ultrasound allows monitoring of the response to endovascular and surgical treatment. This review aims to detail the anatomy and pathogenesis of penile trauma, to give guidance on optimising the contrast enhanced ultrasound technique and to obtain diagnostic examinations. Illustration of the spectrum of aspects of penile trauma will be complemented with a literature review.

**Critical relevance statement** Contrast enhanced ultrasound is a safe and efficient imaging modality that allows for highly accurate diagnoses, helping achieve more successful and conservative treatments of penile trauma complications.

**Key points**

• Ultrasound is an accurate and safe imaging test for penile trauma.

• CEUS adds dynamic vascular imaging in real-time increasing diagnostic confidence.

• CEUS can delineate penile haematomas, locate fractures and demonstrate vessel integrity.

• CEUS potentially allows for more conservative treatment of penile trauma complications.

**Graphical Abstract:**

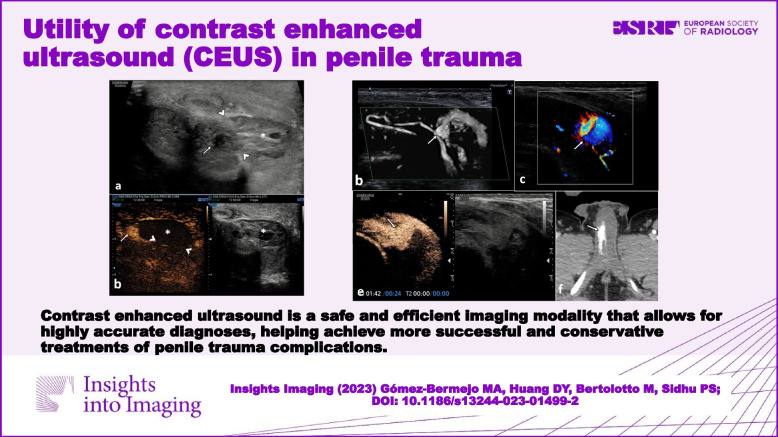

**Supplementary Information:**

The online version contains supplementary material available at 10.1186/s13244-023-01499-2.

## Background

Penile traumatic injuries are rare emergencies with severe life-changing consequences if not managed appropriately. These injuries are often under-reported, as patients may not seek medical care for both ethical and psychological causes [1]. A delay or an erroneous diagnosis could result in ischaemia, infarction, infection, penile atrophy, curvature and consequent infertility [2].

Clinical evaluation following penile trauma can be challenging due to pain and inflammation. Imaging is the basis for diagnosing traumatic penile trauma, allowing a more accurate assessment of the injuries for a more informed decision on either a surgical or a conservative management approach.

Ultrasound (US) is the primary imaging modality for the evaluation of the penis, as it is superficially located. Ultrasound has many advantages over other imaging techniques: high spatial resolution, affordable, readily available, mobile and can be carried out with no patient preparation [2]. Contrast enhanced ultrasound (CEUS) is an advanced ultrasound technique that provides real-time dynamic vascular imaging without exposure to radiation or the need to administer iodinated contrast. The use of CEUS as an adjunct to conventional US examinations is now well-established with a growing range of clinical indications [3].

The aim is to review the sonographic anatomy of the penis, the spectrum of sonographic findings when evaluating penile trauma, and the value added by using CEUS.

## Main text

### Anatomy of the penis (Fig. 1) [2, 4]

**Fig. 1:**
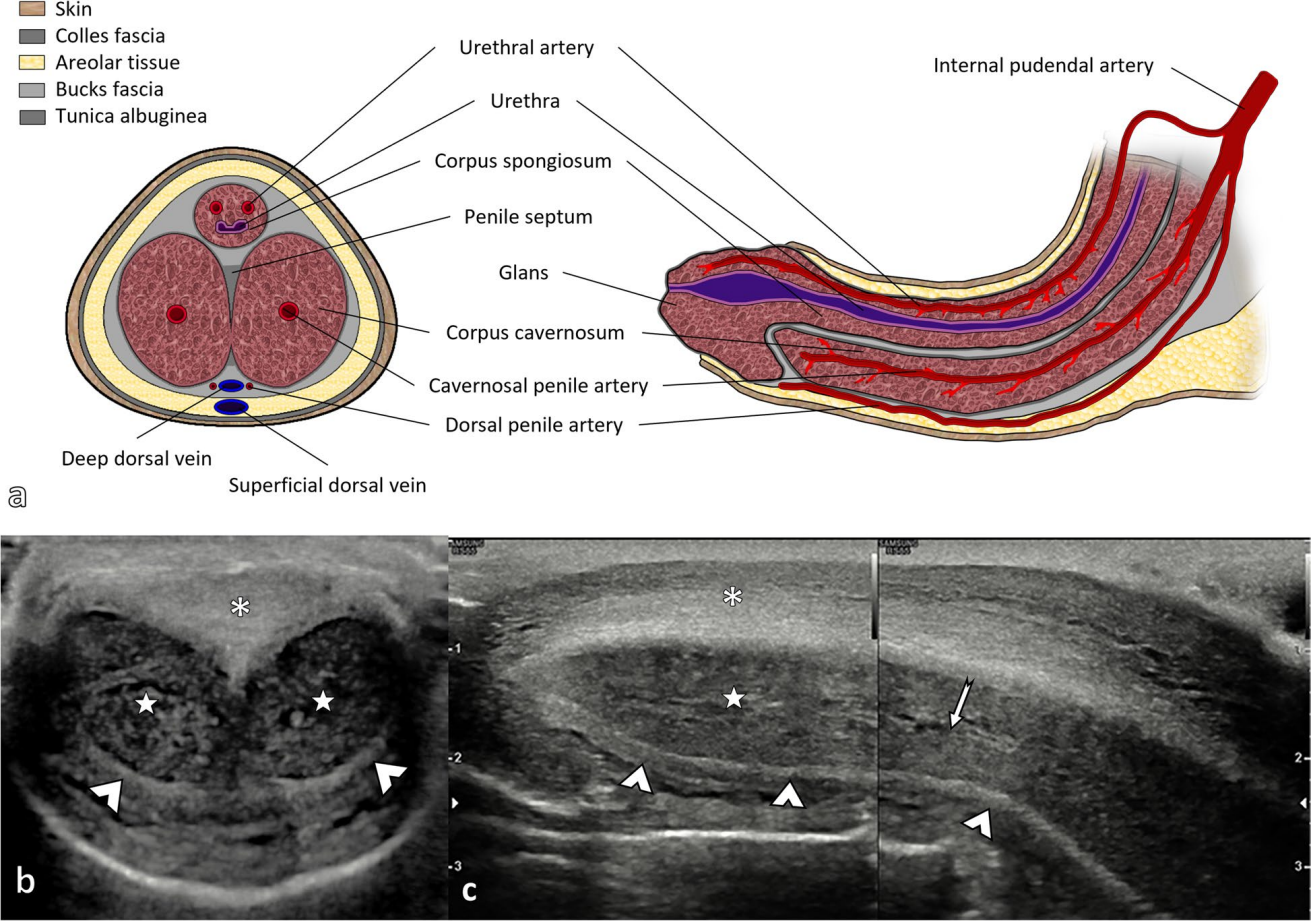
Sectional anatomy of the penis. Anatomical illustration of the penis in the axial plane of the shaft (left) and in the parasagittal plane (right) (**a**). The ultrasound anatomy of the non-erect penis is shown (**b**, **c**) in slices equivalent to those shown in **a**. The corpus spongiosum is in a ventral position (asterisks *), partially collapsed by the weight of the transducer. The corpus spongiosum forms the glans at the distal end of the penis, as seen in the longitudinal view (**c**). The corpora cavernosa (stars) are in a dorsal position. They are all surrounded by a hyperechogenic layer: the tunica albuginea, whose cavernous component is indicated by arrowheads. The arrow indicates the course of the cavernous artery of the penis, which runs in the centre of the corpus cavernosum

The penis comprises two corpora cavernosa on the dorsal surface and a ventral corpus spongiosum on the midline. The cavernosal crura insert into the ischial tuberosities. The corpora cavernosa is constituted by venous sinusoids. During an erection, they are filled with blood. The tunica albuginea encloses both corpora cavernosa. The corpus spongiosum wraps the urethra and forms the glans anteriorly, which is also surrounded by its tunica albuginea. The tunica albuginea is the deepest fibrous layer and encompasses each corpus separately. The superficial fascia of Colles and the deeper fascia of Buck encircle the three corpora. The dorsal artery of the penis runs parallel to the dorsal vein and feeds the glans penis and skin. The cavernosal arteries are located in the centre of the corpus cavernosum (one in each corpus), and they are a terminal branch of the internal pudendal artery. They provide arterial flow during erection. The posterior corpus spongiosum and the urethral bulb are supplied by the bulbourethral artery. The venous supply is made by the dorsal veins, which are the deep dorsal vein (deep to the Buck fascia), and the superficial dorsal vein (above Buck’s fascia). The corpora cavernosa blood is drained through small emissary veins, which reach the cavernosal, dorsal and crural veins.

### Pathogenesis

Penile traumas can be broadly classified into blunt and penetrating injuries. The penis, thanks to its consistency, mobility and location, is protected from injury. It is hence prone to injury during erection [5]. The most frequent aetiology in the Western world is an injury during coitus [6]. In Middle Eastern countries, cases of self-harm to stop an erection are not uncommon [7]. Blunt trauma to a non-erect penis causing a penile fracture is extremely rare. However, it is possible to find vascular injuries due to stretching and compression, as well as intra-cavernous and extra-tunical haematomas [8]. Association for the Surgery of Trauma (AAST) injury score aims to classify penile trauma according to its severity (Table 1) [9].

**Table 1:** American Association for the Surgery of Trauma (AAST) organ injury severity scale for the penis [9]

**Grade**	**Description of injury**
**I**	Cutaneous laceration/contusion
**II**	Bucks’ fascia (cavernosum) laceration without tissue loss
**III**	Cutaneous avulsion/laceration through glans/meatus/cavernosal or urethral defect < 2 cm
**IV**	Cavernosal or urethral defect 2 cm/partial penectomy
**V**	Total penectomy

### Sonographic anatomy of the penis [10]

In the axial view, the two corpora are seen as symmetrical, homogeneous round structures, enclosed by the tunica albuginea, seen as a hyperechoic thin line. The two corpora cavernosa are separated by a septum, which is part of the tunica albuginea. During an erection, the two corpora cavernosa enlarge, and the reflectivity becomes lower. The cavernosal arteries are seen on transverse images as two echogenic dots within the centre of the corpus cavernosum.

### Ultrasound evaluation of penile trauma

#### Patient preparation

In the case of acute penile trauma, the US examination may need to be adjusted to facilitate diagnosis and optimise the patient’s well-being [11]. Testicular and penile injuries are commonly associated, so care should be taken in the positioning of the patient to allow for comfortable sonographic exploration of all the structures. When possible, US examination could be performed with the patient supine while holding his penis by its tip on his abdomen. Placing a supporting towel between the patient’s thighs facilitates better comparative images between the testicles by keeping them elevated and parallel [12]. A careful US investigation using sterile gel and transducer covers must be performed in a degloving injury. The B-mode US is conducted conventionally using a linear transducer ranging between 7 and 15 MHz. Penile US should include longitudinal and transverse grey-scale and Doppler colour flow images of the entire penis. If good visualisation of the base of the penis could not be obtained, a transperineal approach with the elevation of the testes could be considered [2].

#### Contrast enhanced ultrasound

Doppler ultrasound provides information on blood flow by detecting the relative movement of red blood cells compared to the surrounding tissue. However, its capability is limited in the presence of slow flow, moving structures or low haematocrit. CEUS overcomes these limitations by administering UCAs, which are composed of microbubbles that are larger and more reflective than red blood cells, in a fixed amount that allows for increased sound reflection and a better signal [13].

Ultrasound contrast agents (UCA) used in CEUS examination, most commonly Lumason/SonoVueTM (Bracco SpA, Milan), are safe and importantly non-nephrotoxic; no patient preparation is needed, and no pre-examination laboratory testing is required. The phospholipid shell is metabolised by the liver, while the sulphur hexafluoride is eliminated through the lungs [14]. Microbubbles can pass through the pulmonary circulation, remaining in circulation and potentially reaching any vessel in the body. UCA are purely intravascular because they are unable to traverse the vascular endothelium [14]. When the microbubbles are exposed to the ultrasound beam, they resonate in a non-linear way, producing harmonics. This oscillation is best achieved at a resonance frequency of 3–5 MHz. These harmonics make it possible to separate their signal from the echoes reflected linearly by the static tissue, this linear signal being suppressible to obtain specific visualisation images of the UCA. This separation is accomplished with the pulse-inversion technique. CEUS provides information both on a macro- and microvascular level. Compared to the contrast agents currently used in computed tomography (CT) and magnetic resonance imaging (MRI), UCAs have a lower risk of adverse reactions. CEUS is also fast, mobile, cost-effective and can be safely repeatable with multiple injections [3, 11, 15].

#### Penile CEUS technique

We recommend performing the CEUS with a split-screen interface, with B-mode and CEUS images being shown side by side. Low mechanical index (MI) B-mode images (of sub-optimal quality compared to high MI B-mode images) are useful to localise the area of interest. Fusing a contrast-specific image with a low MI B-mode image is also possible. A CEUS examination requires intravenous access for contrast administration. Peripherally inserted 18- to 22-G IV catheters are recommended for injecting the UCA. CEUS has an excellent safety profile [11, 15]. However, there is a low incidence of anaphylactic reactions; therefore, it is advisable to ensure quick access to resuscitation equipment [3].

As a linear high-frequency transducer is used for penile evaluation, a higher dose of 4.8 mL of the SonoVue/Lumason (Bracco SpA, Milan) should be used for achieving optimal enhancement. This is because at higher frequencies the range of sizes of the microbubbles that resonate is less, requiring a higher MI, which results in an accelerated burst of microbubbles.

For a more comfortable examination, it is useful to have an assistant in charge of administering the ultrasound contrast. Before proceeding with the examination, make sure that the ultrasound machine is ready with the settings described above, that a peripheral venous line is placed and the assistant has one syringe of 4.8 mL of SonoVue™ (Bracco SpA Milan) as the UCA and the other with 10 mL of 0.9% normal saline, acting as the flush. At the time of the injection of the UCA by the assistant, the operator should start the timer on the ultrasound machine for recording the examination [3].

#### CEUS technique tips (adapted from [16])

The focus should be just deep to the target, so energy is distributed homogeneously over the imaging plane.

Before injecting the UCA, it is recommended to adjust the gain, so no microbubbles are lost due to non-visualisation when too low gain, or an excess of noise and acoustic shadowing because of signal saturation when too high gain. For reference, the image acquired before the administration of UCA should be nearly black, except for structures with high reflectivity, such as the symphysis pubis.

The dynamic range (the range of signal intensities displayed) should be wide if looking for subtle differences in enhancement, while it can remain low if we want to assess the vasculature itself, ensuring a good contrast of the vessel with the background.

For assessing fast-flowing blood, as in active bleeding, a frame rate of ≥ 10 frames per second is useful. However, it will result in a higher rate of microbubble disruption.

If the MI is set too high, there will be more bubble bursting, especially in the near field, while too low MI leads to worse visualisation of deep structures.

Video clips can be recorded for re-evaluation. This is particularly useful in complex cases.

Continuous scanning of the same area can cause bubble disruption in a very localised area, which can affect the interpretation of the images.

### Sonographic findings of penile trauma

#### Penile haematoma

Penile haematomas can be cavernosal, septal or extra-tunical.

##### Cavernosal haematomas (Fig. 2)

Usually, results from injury to the sub-tunical venous plexus or smooth-muscle trabecula secondary to compression of the penis shaft against the pelvic bones [8, 17]. They are often bilateral and can be associated with penile fractures and arterial injuries with high-flow priapism.

CEUS allows a clear delineation of the borders of the haematoma, which may be obscured on B-mode imaging. If the tunica albuginea is intact, the patient can be treated conservatively.

**Fig. 2:**
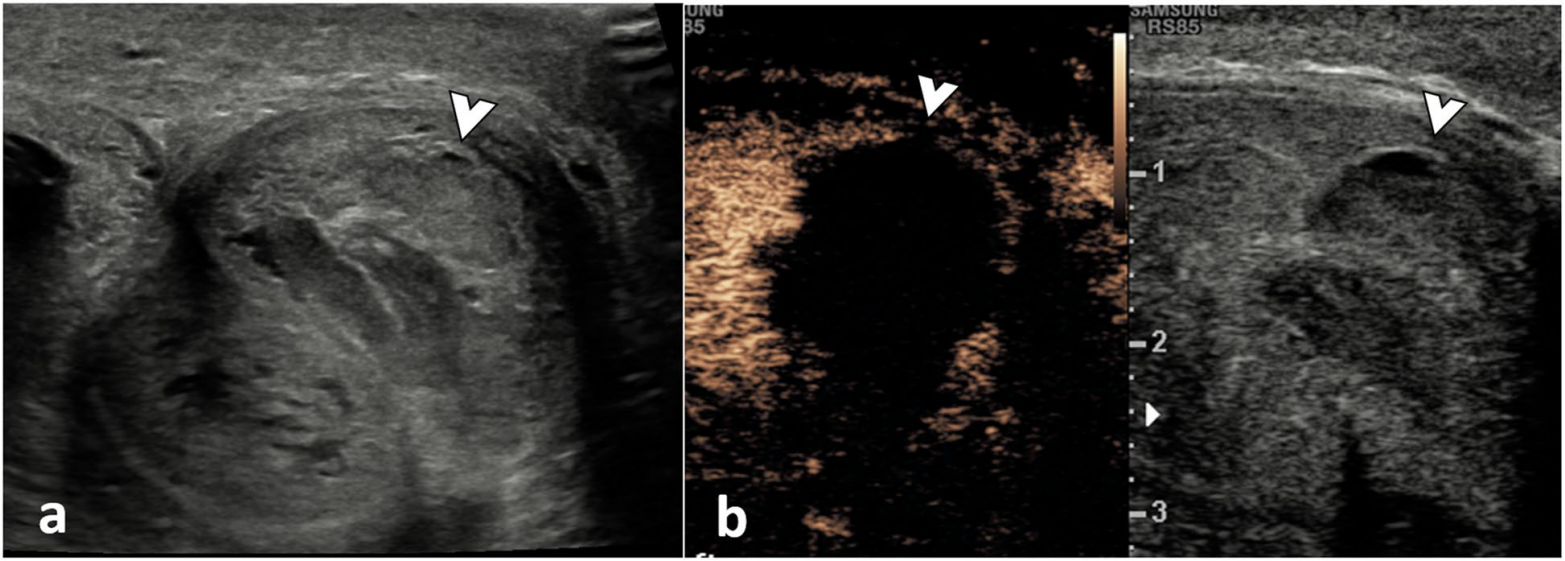
Corpora cavernosal haematoma. A 34-year-old male presented after a high-energy traffic accident with swelling and deformity of the penis. **a** On the B-mode US in an axial plane at the base of the penis, there is an enlargement of the left corpus cavernosum due to an area of mixed reflectivity (arrowhead). Following UCA injection (**b**) the dual aspect view (low MI B-mode image on the right) demonstrates no enhancement of the area on the CEUS image (arrowhead). These findings are in keeping with a cavernosal haematoma. There is no active contrast extravasation nor tears of the tunica albuginea. Note that CEUS allows a clear delineation of the borders of the haematoma, which may be obscured on B-mode imaging alone

##### Septal haematomas (please see Additional file 1: Fig. S1)

Injury to the erect penis can produce isolated disruption of the penile septum, resulting in a haematoma with classical characteristics on US and CEUS. Aspiration under US guidance is recommended to prevent circumscribed septal fibrosis and subsequent penile curving and shortening [18].

##### Extra-tunical haematomas (Fig. 3)

These are secondary to rupture of the dorsal penile vessels and can be either superficial or remain under Buck’s fascia, depending on the site of involvement of the penile veins. It can be associated with an arterial lesion (such as an arteriovenous fistula) or thrombosis of the superficial and deep dorsal penis veins, which is a urological surgical emergency.

The B-mode appearance of a penile haematoma varies with age. In the acute phase, a haematoma is typically hyperechoic. In chronic stages, it becomes a cystic abnormality often with septation [19]. In the long-term, fibrosis can develop, which at US appears as an ill-defined echogenic scar [20].

On CEUS, a penile haematoma appears as a non-enhancing area, corresponding to the B-mode abnormality. CEUS allows a confident diagnosis and a better delineation of the haematoma [21]. The absence of contrast extravasation improves the operators’ confidence in excluding an underlying vascular injury [21].

**Fig. 3:**
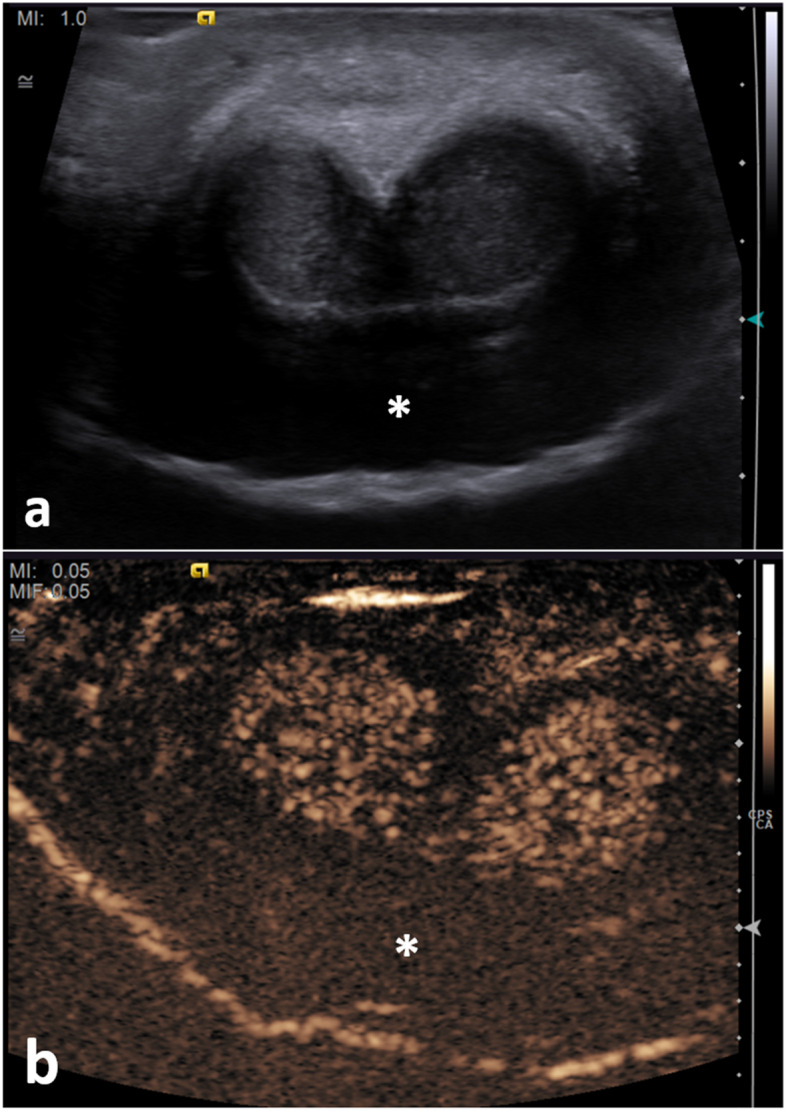
Diffuse venous subacute haematoma. A 26-year-old male who heard a ‘snap’ during intercourse, followed by swelling and pain for 4 days. An US (**a**) and CEUS (**b**) were performed, showing a semi-circumferential hypoechogenic area (asterisk *) present in the subcutaneous tissue of the dorsal aspect of the penis. This area shows no enhancement following the UCA administration. There is the preservation of the integrity of the tunica albuginea on both B-mode (**a**) and CEUS (**b**). Appearances suggest a diffuse venous subacute haematoma

#### Penile rupture (Figs. 4, 5, 6 and 7)

**Fig. 4:**
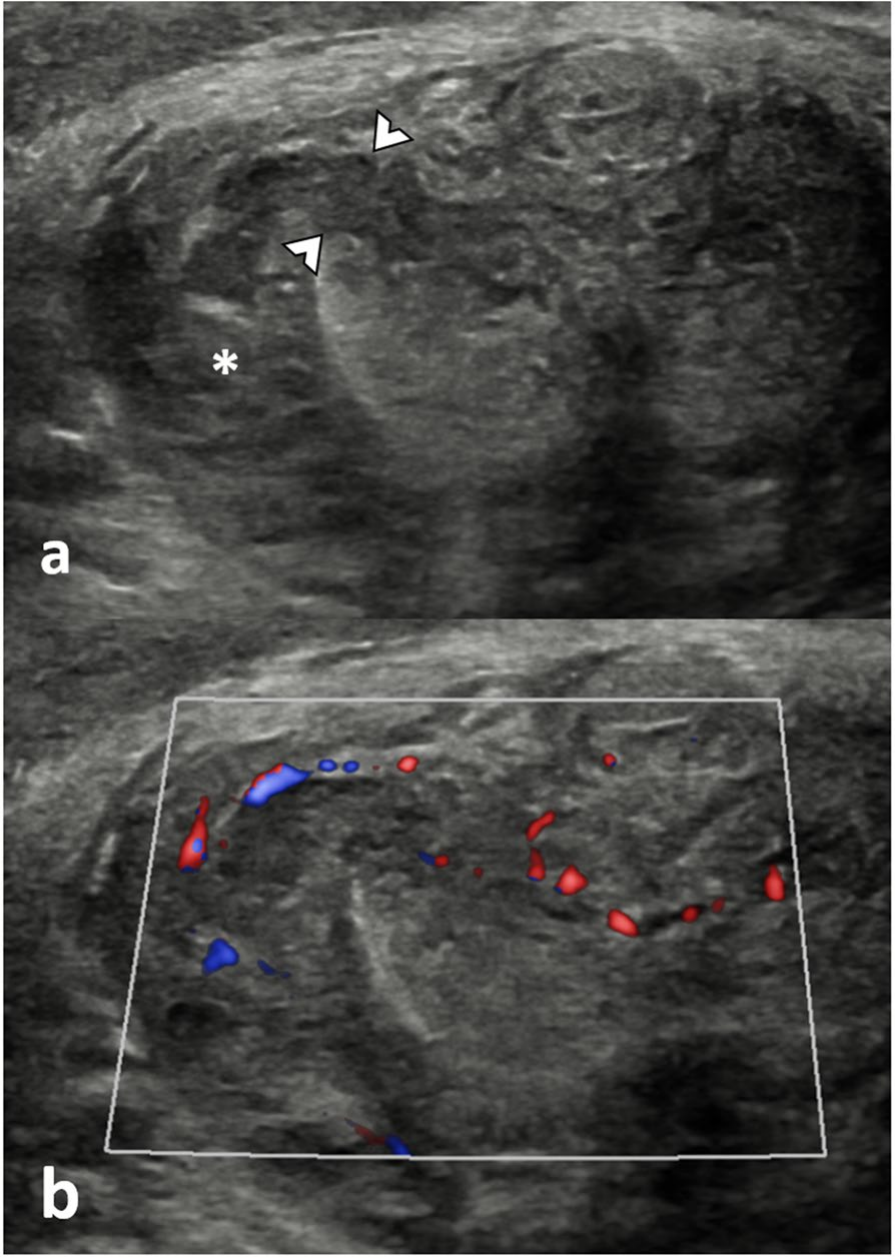
Penile fracture on B-mode and colour Doppler ultrasound. A 33-year-old patient during intercourse twisted and heard a ‘snapping’ sound, followed by rapid detumescence and swelling. An US examination was performed, and a B-mode axial section (**a**) showed a loss of continuity of the tunica albuginea (between arrowheads) of the right corpus cavernosum consistent with a penile fracture and rupture of the corpora cavernosum, associated with a haematoma (asterisk *). The haematoma has internal vascularisation on the colour Doppler study (**b**) corresponding to branches of the dorsal artery which are probably the origin of the haematoma. This example illustrates cases where dehiscence of the tunica albuginea is evident in B-mode US

**Fig. 5:**
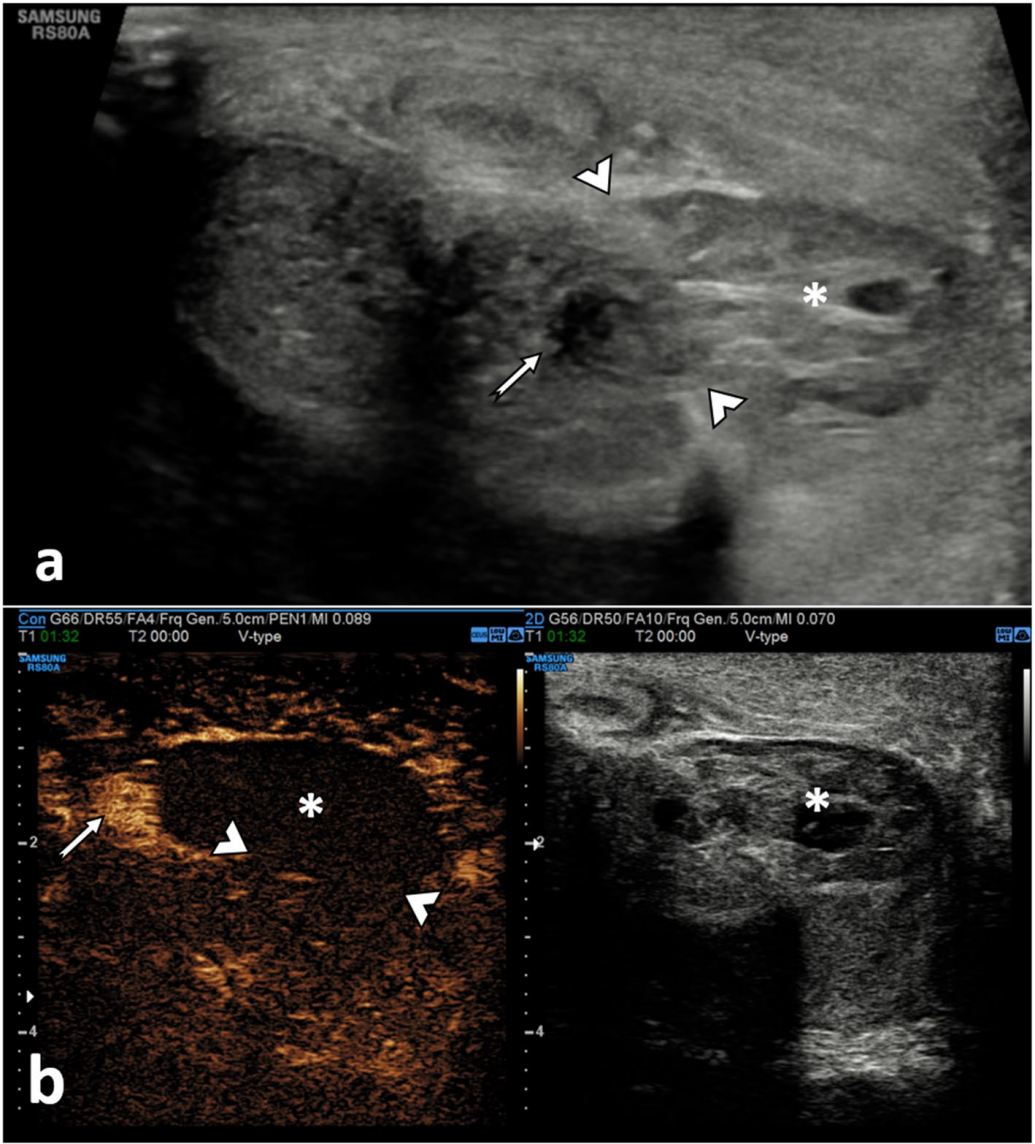
Penile fracture. Haematoma with active bleeding. A 43-year-old man presented with blunt trauma to the penis during sexual intercourse. B-mode US (**a**) demonstrated an area of interruption of the tunica albuginea (between arrowheads) of the left corpus cavernosum with a focal outpouching with mixed echogenicity (asterisk *) extending from the point of rupture. There is a low reflectivity round structure at the site of rupture (arrow). A CEUS was performed (**b**), confirming with more precision the site of the rupture of the tunica albuginea (between arrowheads). The focal outpouching that does not enhance corresponds to a haematoma. The low-reflectivity area seen in B-mode (arrow) corresponds to active extravasation spreading from the site of rupture of the tunica. CEUS allows the detection of the precise location of the rupture, the differentiation of a haematoma from herniated cavernosa parenchyma and the diagnosis of a coexistent active contrast extravasation (arrow). The site of rupture was marked over the skin, allowing for a conservative surgical approach

**Fig. 6:**
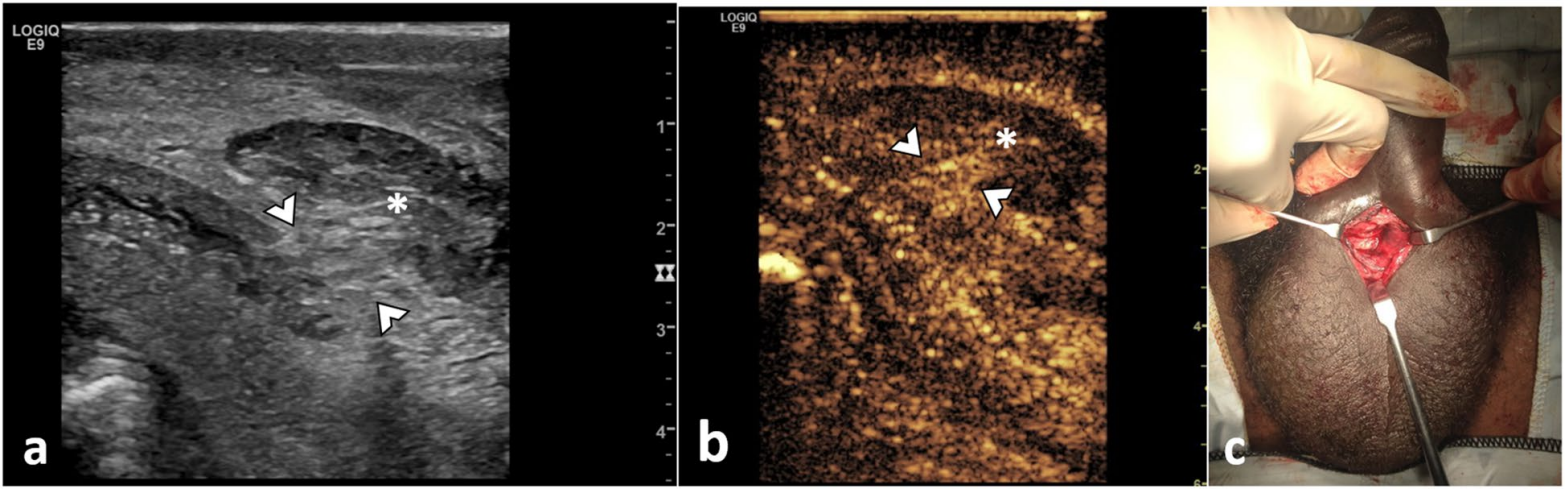
Penile fracture. Haematoma with herniation of corpora cavernosa. A 43-year-old man presented with blunt trauma to the penis during sexual intercourse. US examination reveals an interruption of the tunica albuginea (between arrowheads in **a** and **b**). Superficial to the tunica defect, there is a hypoechoic area (asterisk *), avascular on CEUS, consistent with haematoma. At the rupture site, there is enhancement suggestive of partial herniating adjacent corpora cavernosa. The point of the rupture of the tunica albuginea was marked on the patient’s skin. The intraoperative clinical image (**c**) demonstrates the point of rupture, which exemplifies the degree of minimal invasiveness achievable in surgery guided by US findings

**Fig. 7:**
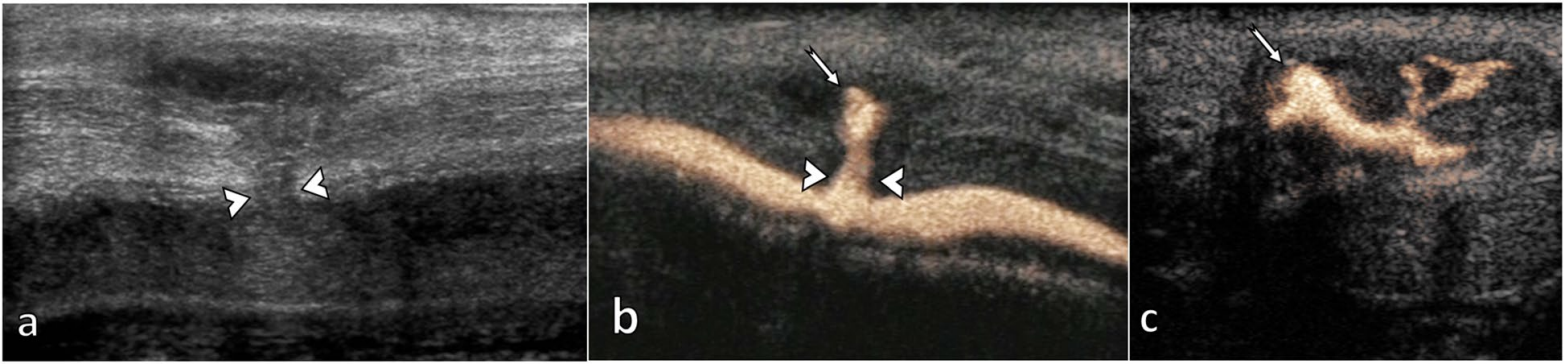
Penile fracture associated with urethral disruption. The patient had a penile injury during intercourse 20 days before but did not present in the hospital. He recovered erections, but eventually presented complaining of persistent bleeding during urination and ejaculation. A small rupture of the tunica albuginea is seen (between arrowheads) on B mode US (**a**). There is a deformation of the corpus spongiosum in the same area, with a fluid collection (arrow) which fills with contrast at contrast sonourethrography in a sagittal (**b**) and axial (**c**) planes

Patients with penile fractures often report hearing a cracking or popping sound at the time of injury, followed by pain and quick reduction of the erection, swelling and discolouration, resulting in the commonly described ‘aubergine deformity’. Penile fractures consist of a tear in the tunica albuginea, typically in the mid or proximal shaft. A penile rupture is considered a urological surgical emergency and requires immediate surgical exploration [2]. In approximately 10–20% of the patients, there is also an injury to the penile urethra. It should be suspected if there is blood in the urethral meatus or in cases of bilateral cavernosal injury [22]. A retrograde ‘intra-cavitary’ CEUS urethrogram [23] (Fig. 7) would be confirmatory for a urethral injury [24].

Ultrasound can detect the exact location and size of the disruption of the tunica albuginea, seen as a tear of the hyperechogenic line surrounding each corpus cavernosum, and herniation of the cavernosal parenchyma if present. Haematomas are commonly present at the fracture site, and in rare cases, a focus of active bleeding may be detected on CEUS [2, 8]. Preoperative US can be useful for surgical planning, allowing for more conservative surgery rather than extensive exploratory intervention [10, 21]. Ultrasound is also of great value in assessing possible complications after the surgical repair of the penile rupture [25].

Contrast enhanced ultrasound provides a confident evaluation of the site of rupture of the tunica albuginea when oedema and bleeding hinder visualisation on B-mode images, confirming the extent and detection of vascular complications. A tear of the tunica albuginea is shown as a non-enhancing area interrupting the linear echogenicity of the tunica [26].

Often a heterogeneous hyperechoic abnormality is found next to the rupture site, which raises the differential between a cavernosal parenchyma herniation or the presence of a haematoma. On CEUS, a herniated cavernosal parenchyma is depicted as an enhancing area continuous with the orthotopic cavernosal corpus, whereas a haematoma will not enhance [3]. Moreover, with its ability to image in real time, CEUS may be able to depict active contrast extravasation, by implication active bleeding, and its origin [21].

#### Vascular injury

##### Cavernosal artery injury (Figs. 8, 9 and 10)

In both penetrating and blunt penile traumas, an injury to a cavernosal artery can occur. This leads to intra-cavernous bleeding, with the patient presenting with high-flow priapism, typically painless. As the venous outflow is preserved, there is a much lower risk of ischaemia. Ultrasound evaluation achieves a precise diagnosis and guides focal compression, embolisation or surgery if needed.

On US, there is usually a haematoma adjacent to the cavernosal artery. When a vascular injury is present, there is an abnormal contour in the B-mode images, with wall discontinuity and/or focal outpouching if there is a pseudoaneurysm. On colour Doppler mode the classic ‘yin-yang’ appearance may be found [27].

On CEUS, the haematoma will appear as a non-enhancing area. CEUS allows clear visualisation of the vascular lumen and demonstrates confidently active extravasation and the presence of pseudoaneurysms [21].

**Fig. 8:**
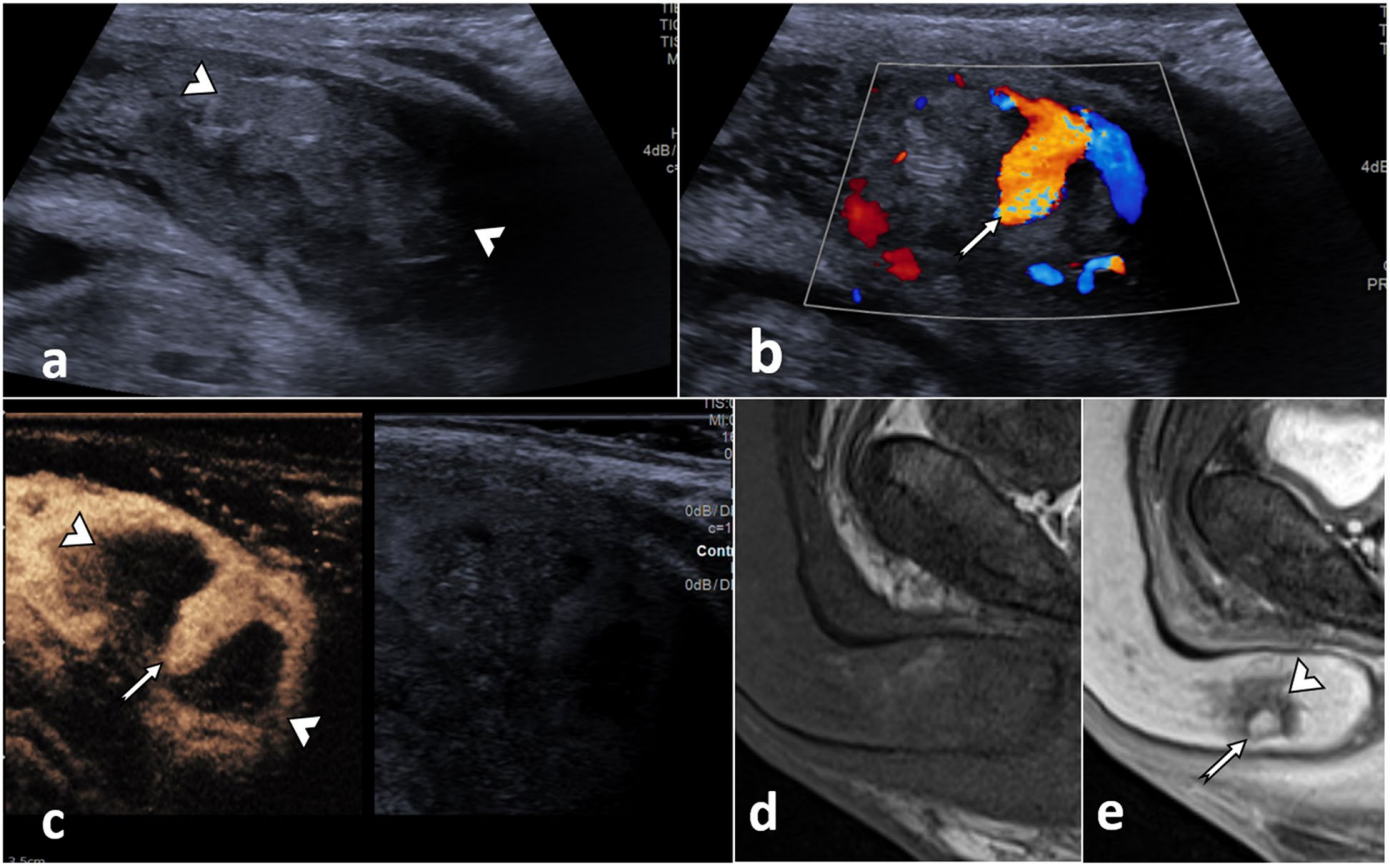
Penile haematoma with pseudoaneurysm. A 38-year-old man presented to the emergency department following a pedal bicycle accident with blunt trauma to the non-erect penis, with clinical high-flow priapism at presentation. Ultrasound was performed. In a sagittal plane in B mode (**a**) where an ill-defined area of mixed echogenicity (between arrowheads) in the right corpus cavernosum at the base of the penis, suspicious of haematoma is seen. The colour Doppler US (**b**) shows a deformity of a branch of the cavernosal artery (arrow) with turbulent flow in its interior, consistent with pseudoaneurysm. CEUS (**c**) confirms the presence of the non-enhancing haematoma (between arrowheads) and the pseudoaneurysm, which enhances avidly (thin arrow) in the arterial phase. A complementary MRI was performed (sagittal T1 pre-contrast (**d**), sagittal T1 FSE C+ (**e**)), showing an area of hyperenhancement of the right corpus cavernosum (arrowhead), compatible with the known haematoma. Within the haematoma, there is a hyperenhancing saccular area corresponding to the pseudoaneurysm (arrow). Note the higher spatial resolution of US compared to MRI in assessing the penis

**Fig. 9:**
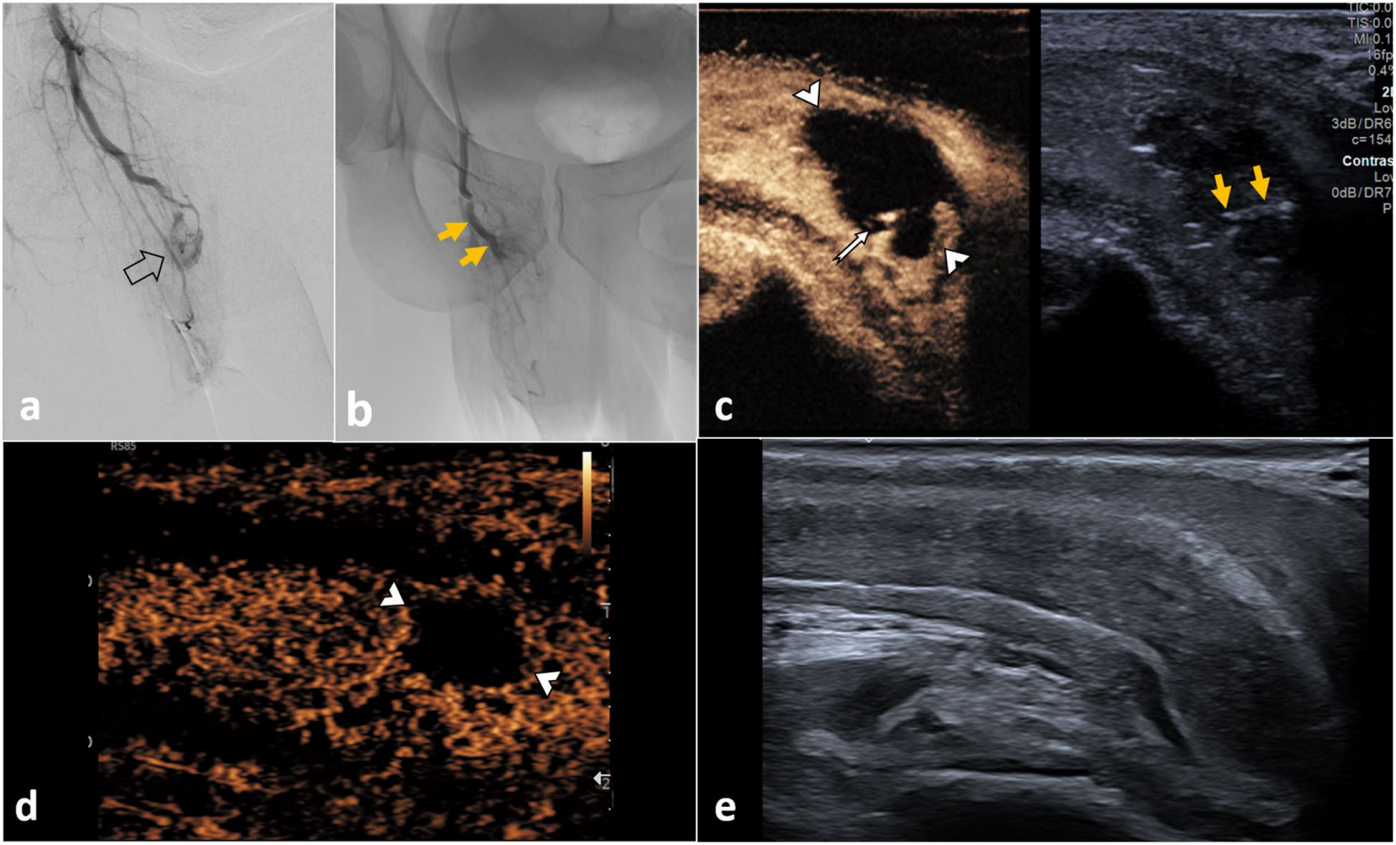
Penile haematoma with pseudoaneurysm treated (same case in Fig. 8). Selective arteriography of the right cavernous artery was performed (**a**), showing the pseudoaneurysm with active bleeding (open arrow). Supra-selective embolisation was performed with a gelatin sponge (yellow arrows) (**b**). CEUS is performed immediately after the procedure (**c**), illustrating the haematoma (between arrowheads). There is now the absence of the previously documented pseudoaneurysm with a residual stump noted (arrow). The hyperechogenic material (yellow arrows) visible on the low MI B-mode image is the embolisation gelatin sponge material. The patient’s priapism resolved within 24 h post-embolisation. CEUS 2 weeks after the procedure (**d**) shows a decrease in the size of the haematoma (between arrowheads) with no pseudoaneurysm visible. One and a half months after embolisation (**e**), the appearance of the penis on ultrasound is normal. The patient reported a fully recovered erectile function 8 weeks post-embolisation

**Fig. 10:**
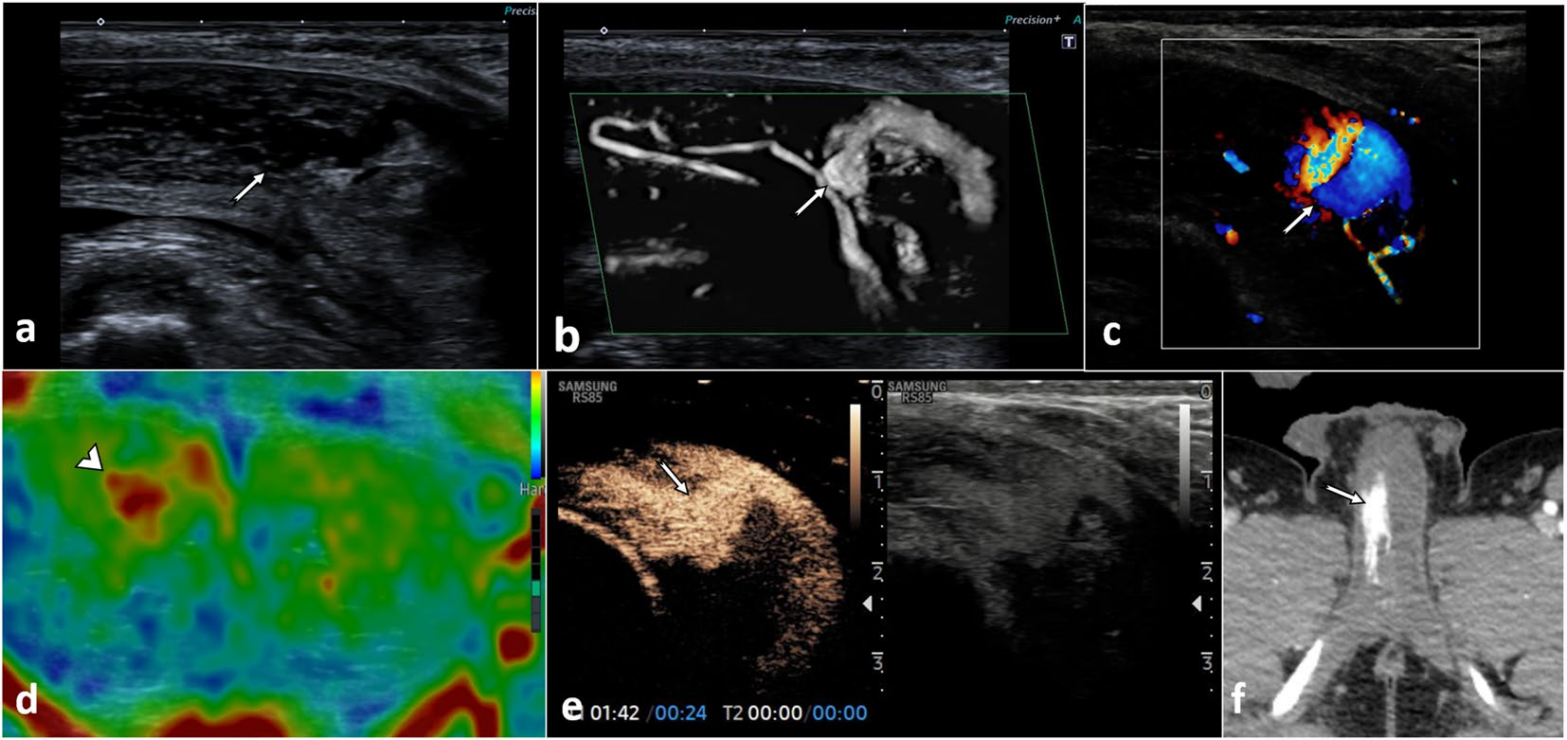
Cavernosal haematoma with an injury of the cavernosal artery and an arterio-lacunar fistula. A 26-year-old patient attended the emergency department for priapism after a perineal injury caused by a fall from a pedal bicycle. An emergent US was performed, showing in the sagittal plane in B-mode (**a**) and in microvascular imaging (MVI) mode (**b**) an abnormality of the proximal aspect of the right cavernous artery (arrow) with an outpouching, suggestive of pseudoaneurysm is demonstrated. On colour Doppler mode (**c**), the ‘yin-yang’ sign (arrow) is identified, representing circumferential flow within a pseudoaneurysm. In addition, elastography (**d**) shows a greater rigidity of the right corpus cavernosum (arrowhead), probably due to the higher blood content compared to the left corpus cavernosum. After administration of UCA (**e**), the injury to the cavernous artery (arrow) is demonstrated, as well as contrast extravasation and early filling of that portion of the right cavernous body, compatible with an arterio-lacunar fistula. The CT scan performed for polytrauma in the emergency department (**f**) shows early enhancement of the right corpus cavernosum compared to the left, as an indirect sign of an arterio-lacunar fistula

##### Traumatic thrombosis of corpus cavernosum (Fig. 11)

Repetitive perineal compression, such as in bicycle users, can lead to partial thrombosis of the corpus cavernosum. Its incidence is increased in proliferative, coagulation and haematological disorders and when there is the use of drugs or medications such as tamsulosin or sildenafil. The thrombus is commonly unilateral and proximally located. The presence of a fibrous connective tissue septum at the cruro-cavernous junction has been associated with its development [28].

Ultrasound shows a swollen, non-compressible corpus cavernosum with the absence of vascularisation in CEUS.

**Fig. 11:**
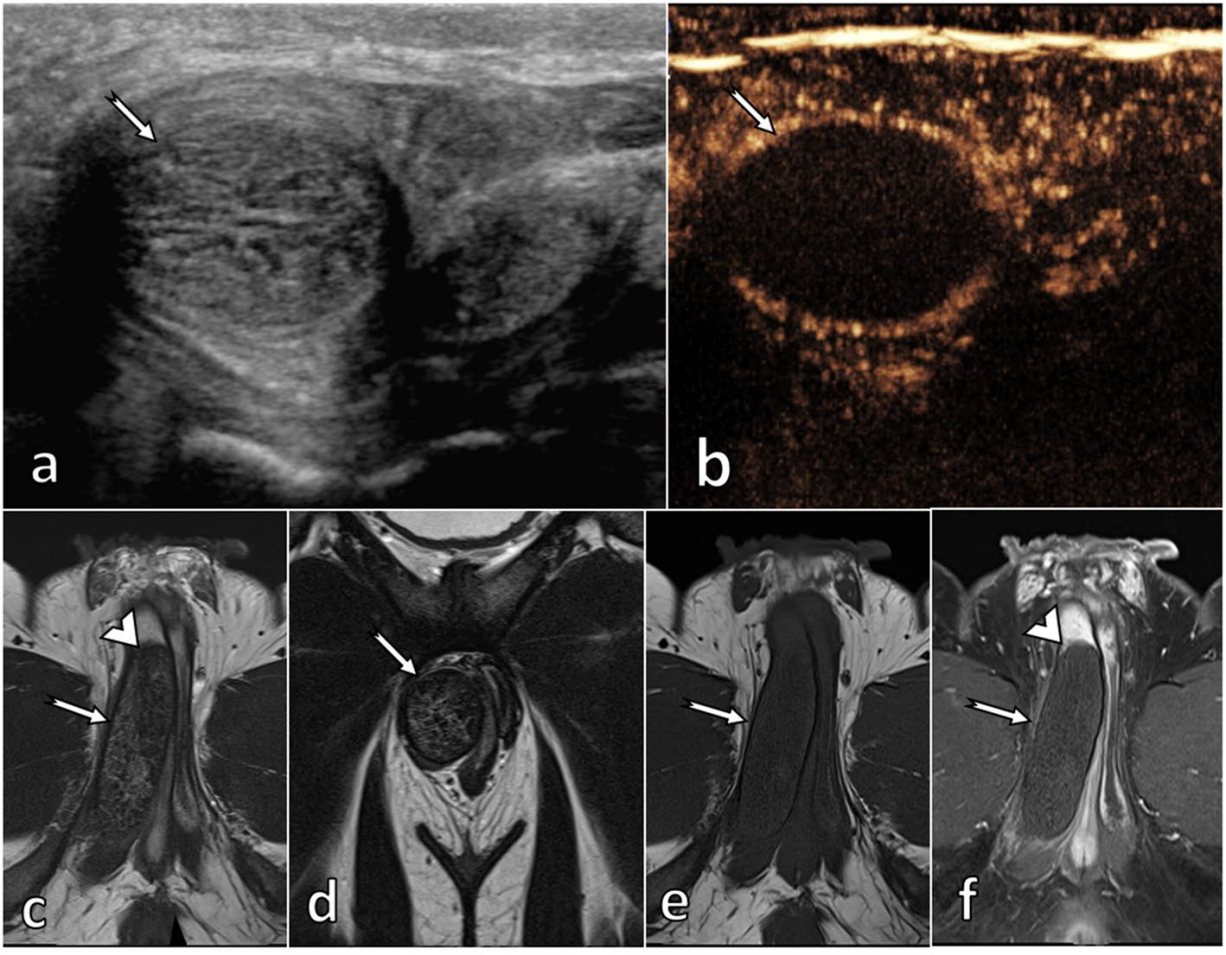
Partial segmental thrombosis of the corpus cavernosum. A 51-year-old patient, an avid mountain biker, developed a painful palpable lump in the perineum after several hours of riding his bike. US (**a**) shows a swollen, non-compressible right corpus cavernosum (arrow), lacking vascularisation at CEUS (**b**), consistent with partial segmental thrombosis of the corpus cavernosum. Diagnosis is straightforward considering history (repeated perineal microtraumas) and US features. Confirmatory MRI, however, has been done, showing a hypointense on T2WI (**c**, **d**), non-enhancing (**e**, **f**) thrombus in the crus of the right corpus cavernosum which is divided into two parts by a web (arrowhead). This is a well-known predisposing factor for partial segmental thrombosis

##### Traumatic thrombosis of the dorsal veins

Thrombosis of the superficial and deep dorsal penile veins is a urological surgical emergency. It can be secondary to trauma or strenuous sexual activity. It may present clinically similar to penile fracture.

B-mode US shows a non-compressible dorsal vein without internal signal on colour Doppler US [2]. In difficult cases, CEUS can help confirm or exclude thrombosis with the demonstration of the presence of microbubbles within the vessel lumen.

##### Dorsal vein rupture

These injuries can mimic penile fractures. Rupture of small venous collaterals is more common than injury to the main branches.

In most cases, torn veins are not visible directly on US as they are collapsed. It can sometimes be associated with a traumatic arteriovenous fistula, showing an alteration in the morphology of the affected vessels and the presence of pulsatility in the vein. Doppler US reveals low-resistance high-velocity arterial flows and turbulent high-velocity venous flows [8].

#### Traumatic dislocation of the penis—‘buried penis’ (Fig. 12)

Penile dislocation following pelvic trauma is very rare. It is generated by a high-energy impact on the pelvis, causing a displaced fracture with dislocation of the pubic symphysis. The retraction of the pubic bone draws on the penis by its suspensory ligament, retracting violently the penis and causing a circumferential tear around the prepuce with separation of penile skin from the shaft. The penis shaft may be displaced into the inguinal canal, scrotum or subcutaneous tissue in front of the pubis, depending on the direction of the pull. The patient may present with a suspected amputation of the penis, so the dislocation remains unnoticed [29, 30].

Ultrasound may be useful in identifying a dislocated penis. If the exploration is inconclusive, an MRI would be definitive in confirming or ruling out a penile dislocation [29].

CEUS may be useful to confirm the ectopic presence of penile tissue and its viability. A non-enhancing dislocated penis may be ischaemic and lead to surgery with a non-reconstructive intention.

**Fig. 12:**
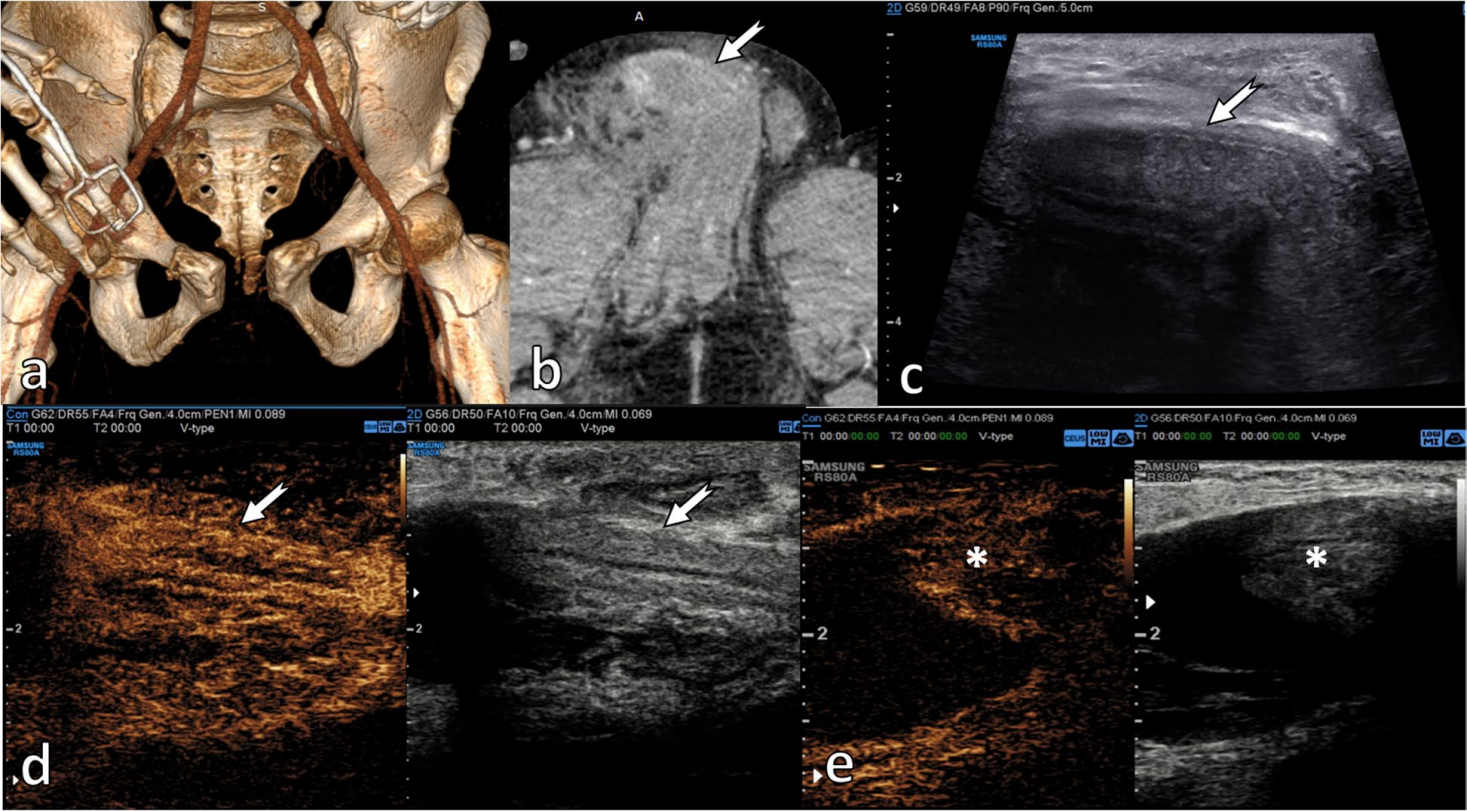
Traumatic dislocation of the penis. A 26-year-old male patient, riding a moped, who suffered polytrauma following a collision with a motor vehicle. Injuries included a fracture of the left superior and inferior pubic rami, left acetabulum and pubic symphysis diastasis, as illustrated in the 3D surface reconstruction (**a**). In addition, the patient was clinically diagnosed with traumatic penile amputation, with only a remnant of skin remaining. The initial CT examination did not observe the intact penis which was dislocated and ‘buried’ in the soft tissues of the groin area (arrow in (**b**)), towards the right inguinal canal. Following a 1-week intensive care admission, the patient reported a pubic lump with intermittent hardening. An US examination was performed, demonstrating on B-mode (**c**) the presence of the penis under the skin (thin arrow). On CEUS, there was preserved vascularisation in both the shaft (arrow) (**d**) and the glans penis (asterisk *) (**e**), indicating viability. The patient successfully underwent surgery and was able to regain a fully functional erectile response

## Conclusions

Penile trauma is a urological emergency often underdiagnosed and can lead to severe complications, requiring rapid evaluation both clinically and by imaging. Multiparametric US is an ideal diagnostic tool in penile traumatic injuries, with high resolution, is ready availability at the patient’s bedside with a good safety profile. Contrast enhanced ultrasound provides greater diagnostic accuracy and certainty to guide the treatment of traumatic penile injuries, improving the efficacy of baseline US techniques.

### Supplementary Information

**Additional file 1:** **Fig. S1.** Isolated septal haematoma. A 47-year-old patient experienced acute pain after accidental bending of the erect penis during intercourse. The penis remained rigid, but a palpable lump appeared after the erection, painful at palpation. An anechoic lesion was found at US in the cavernosal septum (arrow in a), lacking vascularisation at CEUS (arrow in b), consistent with isolated septal haematoma. Blood content is confirmed at MRI, being isointense to corpus cavernosum on T2WI (arrow in c), hyperintense on fat-saturated T1WI (arrow in d), without changes in signal on post-contrast T1WI (arrow in e) confirmed on subtraction images (arrow in f). Please note that the patient has Peyronie’s disease (unaware before the trauma), a predisposing factor for this injury.

## Data Availability

Data sharing is not applicable to this article as no datasets were generated or analysed during the current study.

## Abbreviations

AAST: American Association for the Surgery of Trauma
CEUS: Contrast enhanced ultrasound
CT: Computed tomography
MI: Mechanical index
MRI: Magnetic resonance imaging
UCA: Ultrasound contrast agent
US: Ultrasound
