# Supplementary material for: Utility of contrast enhanced ultrasound (CEUS) in penile trauma
Source: Insights Imaging. 2023 Sep 25;14:158. doi: 10.1186/s13244-023-01499-2 (PMC10519907; doi:10.1186/s13244-023-01499-2)
Supplement: Supplementary file 1 — Additional file 1: Fig. S1. Isolated septal haematoma. A 47-year-old patient experienced acute pain after accidental bending of the erect penis during intercourse. The penis remained rigid, but a palpable lump appeared after the erection, painful at palpation. An anechoic lesion was found at US in the cavernosal septum (arrow in a), lacking vascularisation at CEUS (arrow in b), consistent with isolated septal haematoma. Blood content is confirmed at MRI, being isointense to corpus cavernosum on T2WI (arrow in c), hyperintense on fat-saturated T1WI (arrow in d), without changes in signal on post-contrast T1WI (arrow in e) confirmed on subtraction images (arrow in f). Please note that the patient has Peyronie’s disease (unaware before the trauma), a predisposing factor for this injury. [file 13244_2023_1499_MOESM1_ESM.docx]

**Utility of Contrast Enhanced Ultrasound (CEUS) in penile trauma**

**ELECTRONIC SUPPLEMENTARY MATERIAL**

**
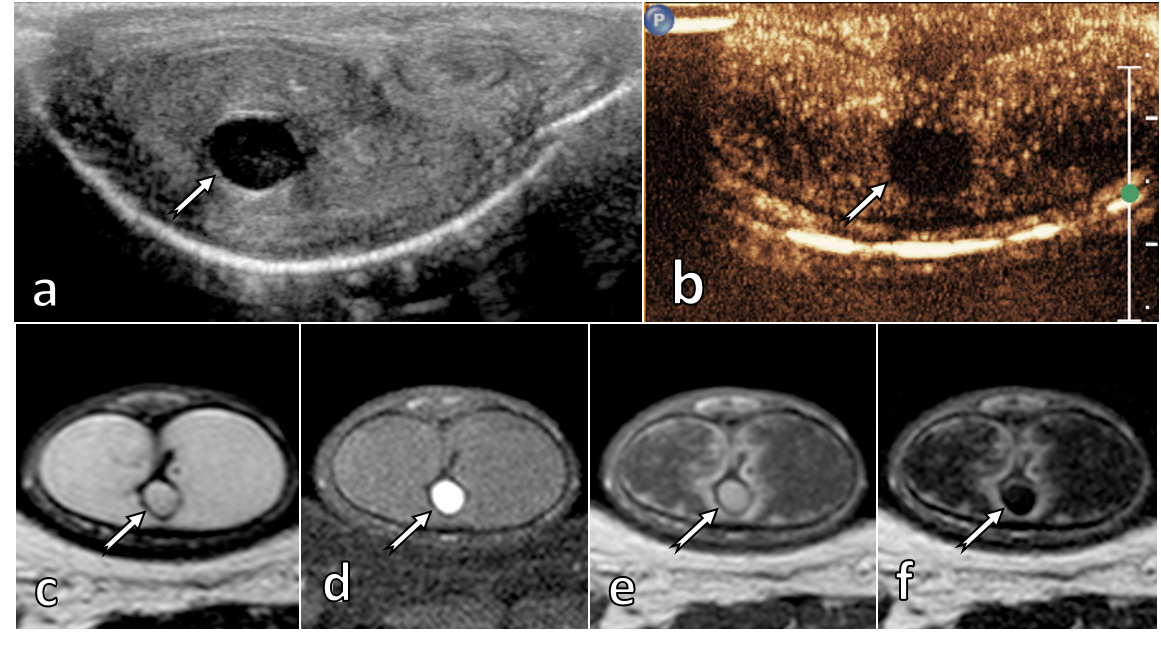
**

**Figure S1.** Isolated septal haematoma.

A 47-year-old patient experienced acute pain after accidental bending of the erect penis during intercourse. The penis remained rigid, but a palpable lump appeared after the erection, painful at palpation. An anechoic lesion was found at US in the cavernosal septum (arrow in a), lacking vascularization at CEUS (arrow in b), consistent with isolated septal haematoma. Blood content is confirmed at MRI, being isointense to corpus cavernosum on T2WI (arrow in c), hyperintense on fat-saturated T1WI (arrow in d), without changes in signal on post-contrast T1WI (arrow in e) confirmed on subtraction images (arrow in f). Please note that the patient has Peyronie’s disease (unaware before the trauma), a predisposing factor for this injury.
